# The effects of physical exercise on anxiety symptoms of college students: A meta-analysis

**DOI:** 10.3389/fpsyg.2023.1136900

**Published:** 2023-03-30

**Authors:** Yanru Lin, Wei Gao

**Affiliations:** School of Physical Education and Sport Science, Fujian Normal University, Fuzhou, China

**Keywords:** aerobic exercise, type of exercise, exercise intensity, psychology, exercise time

## Abstract

**Objective:**

This study aimed to evaluate the effect of an exercise intervention on improving and alleviating anxiety symptoms in college students with a meta-analytical approach.

**Methods:**

Several databases (e.g., PubMed, Embase, and the Cochrane Library) were used to search for randomized controlled trials (in short, RCTs) on interventions for physical exercise or aerobic exercise in college students with anxiety symptoms. Stata software, version 16.0, was applied sequentially for traditional meta-analysis, subgroup analysis, and publication bias analysis.

**Results:**

A total of nine papers were included. The total literature effect [SMD = −0.55, 95% CI = −0.76 to −0.35, *Z* = 5.38 (*P* < 0.001)] indicated that physical activity had a significant effect on alleviating anxiety. Subgroup analysis also showed that exercise interventions using aerobic exercise or yoga were effective in relieving anxiety (SMD = −0.39, 95% CI = −0.74 to −0.04; SMD = −0.76, 95% CI = −1.14 to −0.39).

**Conclusion:**

Physical activity interventions were shown to have a positive effect on alleviating anxiety in college students. Aerobic exercise was found to be the optimal mode.

## 1. Introduction

Anxiety is composed of complex emotional states, such as tension, worry, and uneasiness, that arise from upcoming situations that could pose a danger or threat (Shihua et al., 2016). If anxiety states become severe, they have the potential to develop into undesirable symptoms and eventually become anxiety disorders (Menghuan and Qingqi, 2021). An anxiety disorder is mainly characterized by anxious emotional experiences, which are manifested as fear, restlessness, etc., that can damage the original healthy life and greatly reduce the effect of disease treatment.

Anxiety is becoming a pervasive public health problem that has been associated with unhealthy behaviors, such as lack of physical activity, smoking, and poor diet, subsequently leading to an increased risk of health problems, even among healthy individuals (Bonnet et al., 2005). College students are among the most affected groups of people due to their potentially having to manage their stress, anger, or frustration on their own for the first time (Liu et al., 2022). They must not only face various problems in their studies, work, and feelings but also solve problems of interpersonal communication. Without guidance and support, college students could feel anxious and pressured (Guo et al., 2018). A substantial proportion of anxious college students have sub-health problems due to unhealthy living habits, such as drinking, smoking, staying up late and eating irregularly. During the outbreak of the novel coronavirus, the mental health problems of college students have been reported as becoming more serious, and the physical inactivity caused by the lockdown has caused student’s mental status to become even worse (Rogowska et al., 2020; Xiang et al., 2020).

When college students encounter mental health problems, they tend to solve them on their own, and professional psychological counseling treatment or medication is their last option (Fortney et al., 2016). According to previous studies, current treatments for anxiety disorders include pharmacotherapy with serotonin reuptake inhibitors and cognitive behavioral therapy (CBT) (Taylor et al., 2012), with medication being the most common treatment for anxiety (Barlow, 2004). The effectiveness of medication has been confirmed in treating anxiety disorders, but medication’s side effects are significant. Treatment with medication also has the possibility of relapse after stopping the medication, so it might not be a long-term solution for some patients (Broocks et al., 1998). At the same time, some scholars have demonstrated that exercise can bring many physiological changes that could improve emotional status and reduce stress and anxiety levels (Warburton et al., 2006; Wipfli et al., 2008; Carter et al., 2021). Exercise interventions have become a complementary and alternative method for relieving anxiety. Mind-body exercises are increasingly used as adjunctive and alternative therapies to manage psychological stress. Some studies have also suggested exercise as a possible option for treating anxiety symptoms and anxiety disorders, playing a dual role in adjunctive therapy (pharmacotherapy or psychotherapy) and exercise (Paluska and Schwenk, 2000; Hovland et al., 2013; Mohamed and Alawna, 2020; Wang et al., 2020). However, exercise interventions can only be used as an auxiliary means (in conjunction with other treatments) and cannot be used as the only means to treat psychological disorders.

Exercise interventions are planned and organized with the goal of improving physical fitness and enhancing positive mood. For studies aimed at determining the effects of exercise on mental health, most have preferred a form of exercise that can benefit cardiopulmonary regulation, such as aerobic exercise (Broman-Fulks et al., 2004; Abd El-Kade and Al-Jiffri, 2016). Other forms of exercise, such as yoga and tai chi, have also been found to be effective in improving anxiety (Berger and Owen, 1992; Huston and McFarlane, 2016).

For people with anxiety disorders, exercise can be a treatment option that is promising, affordable and accessible. Numerous observational studies have demonstrated that exercise, especially aerobic exercise, is inversely associated with anxiety symptoms (Larun et al., 2006; Wegner et al., 2014; LeBouthillier and Asmundson, 2015; Stonerock et al., 2015). Both qualitative and quantitative studies have illustrated the effects of aerobic exercise on anxiety symptoms. For example, people who perform physical activity regularly can have higher self-confidence and less anxiety and depression. There have also been studies linking aerobic exercise to cognitive abilities that have confirmed that aerobic exercise can improve cognitive performance (Masley et al., 2009; Stern et al., 2019). Aerobic exercise is effective for people’s cognitive behavior, subjective wellbeing and mental health, and people’s cognitive control and attention are also enhanced after aerobic exercise (White et al., 2017; Pontifex et al., 2019). However, excessive, inappropriate movement can still cause some damage to the body and increase the risk of anxiety. At the same time, few studies have compared the effectiveness of aerobic exercise with that of other exercises in improving anxiety symptoms. Therefore, the main purpose of this study was to explore the efficacy of different modes of exercise as a treatment regimen for anxiety disorders in college students. Additionally, the relationships of different exercise intensities, exercise times and exercise types with anxiety in college students were also determined.

## 2. Materials and methods

### 2.1. Literature search strategies

The literature search used the Boolean logic algorithm to obtain subject words and free words, covering databases including China national knowledge infrastructure (CNKI), Wanfang, Weipu, PubMed, Embase, Cochrane Library, and Web of Science for articles published up to January 19, 2022. The search strategy was generated as follows: [(Anxiety OR Angst OR social anxiety OR Anxieties OR anxiety social OR Hypervigilance OR Nervousness OR Anxiousness) AND (Exercise OR physical activity OR Exercises OR activities physical OR activity physical OR physical activities) AND (randomized controlled trial OR randomized OR placebo)].

### 2.2. Inclusion and exclusion criteria of literature

The literature screening applied population, intervention, comparison, outcome and study design (PICOS) strategies, which have been widely used in evidence-based medicine or practice. It is necessary to specifically identify the acronyms for all or some of the elements in clinical trials (Akobeng, 2005). Specifically, P refers to the research object (participants), I the intervention method (intervention), C the control group (comparison), O the outcome index (out-come), and S the study design. In the current study, the literature inclusion criteria were: (1) the research target was college students; (2) the intervention methods were aerobic exercise, yoga, etc.; and (3) the studies were randomized, controlled experiments. The exclusion criteria were gray literature and review literature.

### 2.3. Literature quality evaluation

This study used the he Physiotherapy Evidence Database (or “PEDro” for short) scale to evaluate the quality of RCTs (Verhagen et al., 1998). Literature quality scoring was conducted independently by two authors. If there was any disagreement, a third author was consulted until a unanimous decision was made.

### 2.4. Statistical methods

The included literature was analyzed using Stata software, version 16.0. The outcome measures in the included literature were continuous variables, which were combined with the effect amounts. The effect indicators were calculated using the standard mean difference (SMD) and 95% CI calculation method. The heterogeneity between the studies was statistically analyzed by I^2^. If *I*^2^ > 50%, the heterogeneity was analyzed by a random effects model. Otherwise (*I*^2^ < 50%), a fixed-effects model was applied. Stata software, version 16.0, was used for bias analysis and subgroup analysis.

## 3. Results

### 3.1. Included meta-analyses

**FIGURE 1:**
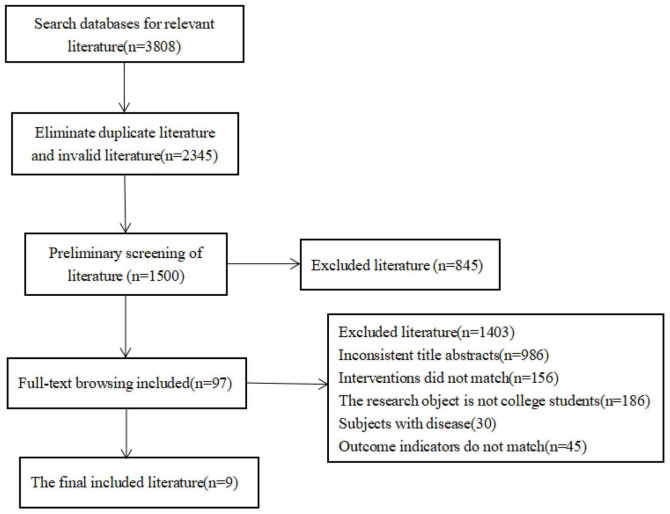
Literature screening flowchart.

The PRISMA flowchart in Figure 1 depicts the specific literature search process. A total of 3,808 relevant documents were obtained through computer searches, and 2,345 were retained after removing duplications. During the screening process, 854 articles were excluded after reading the titles and abstracts. Nine documents were finally included after eliminating interventions that did not include exercises or physical activity, targeted non-university students, or had inconsistent outcome indicators (*n* = 1,491 removed).

### 3.2. Basic features of the included literature

Nine studies and 483 participants were included in the current study, including four aerobic exercise interventions, two yoga interventions, one intervention applying aerobic exercise combined with resistance training, and two other forms. All of the studies included were written in English. Table 1 demonstrates the basic features of these studies, including the intensity and frequency of exercise and the outcome indicators. Specifically, three studies used social anxiety as the outcome indicator, and one used heart rate.

**TABLE 1:** Basic features of the included literature.

Study	Experimental interventions	Sample size	Exercise intensity	Duration	Frequency	Time	Outcomes
Anshel, 1996	Aerobics exercise	60	High	10 weeks	1 time/week	30 min	Heart rate
Albracht-Schulte and Robert-McComb, 2018	Yoga	40	Medium	4 weeks	2 times/week	30 min	STAI rating
Jazaieri et al., 2012	Mindfulness intervention	56	Low	12 weeks	3 times/week	70 min	SIAS rating
Broman-Fulks and Storey, 2008	Aerobics exercise	24	Medium	2 weeks	3 times/week	20 min	ASI-R score
Blough and Loprinzi, 2018	Aerobics exercise	57	High	24 weeks	3 times/week	50 min	PHQ-9
Herring et al., 2012	Aerobic exercise, resistance	30	High	6 weeks	2 times/week	40 min	PSWQ score
Smits et al., 2008	Aerobic exercise	60	High	3 weeks	2 times/week	20 min	ASI, BAI
Tekur et al., 2012	Yoga	80	Medium	1 week	5 times/week	60 min	STAI rating
Tsai et al., 2003	Tai Chi	76	High	12 weeks	3 times/week	50 min	STAI rating

STAI score, Status-Trait Anxiety Scale; SIAS score, Social Anxiety Scale; ASI-R score, anxiety sensitivity index revision; PHQ-9 score, depression screening scale; NIMH-SR, The National Institute of Mental Health Self-Rating Scale; ASI, anxiety sensitivity index; BAI, beck anxiety inventory.

### 3.3. Inclusion in literature quality evaluation

For the literature quality evaluation, all of the included literature achieved the criteria of “random allocation,” “intention-to-treat analysis (ITT) intentional treatment analysis,” “statistical analysis between groups,” and “point measurement and variation value measurement.” For the PEDro scoring, 1 article received 5 points, 7 received 6 points, and 1 received 8 points. The average PEDro score of the included literature was approximately 6 points. The overall methodological quality was adequate (Table 2).

**TABLE 2:** Methodological quality assessment of the included literature using the tool PEDro scale.

Study	Random allocation	Distribute hide	Baseline similar	Subjects were blinded	Baseline similar	Result blind	With- drawal rate < 15%	ITT intentional treatment analysis	Statistical analysis between groups	Point measurements and variance magnitudes	Total
Anshel, 1996	1	0	1	0	0	0	1	1	1	1	6
Albracht-Schulte and Robert-McComb, 2018	1	0	1	0	0	0	0	1	1	1	5
Jazaieri et al., 2012	1	0	1	0	0	0	1	1	1	1	6
Broman-Fulks and Storey, 2008	1	0	1	0	0	0	1	1	1	1	6
Blough and Loprinzi, 2018	1	0	1	0	0	0	1	1	1	1	6
Herring et al., 2012	1	0	1	0	0	0	1	1	1	1	6
Smits et al., 2008	1	0	1	0	0	0	1	1	1	1	6
Tekur et al., 2012	1	1	1	0	0	1	1	1	1	1	8
Tsai et al., 2003	1	1	1	0	0	0	1	1	1	1	6

### 3.4. Meta-analysis results

**FIGURE 2:**
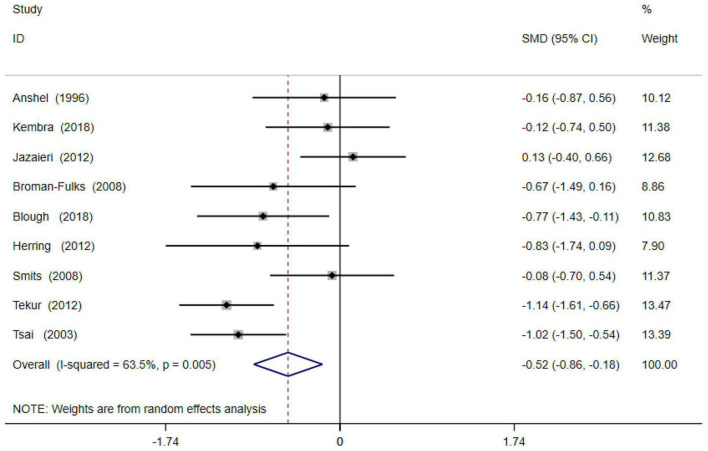
Forest diagram of physical activity and anxiety symptoms.

Forest maps were used for heterogeneity testing. The results showed that there was heterogeneity between the studies (*I*^2^ = 63.5%, *P* < 0.05); thus, a random effects model was adopted for the combined effect size of SMD. The combined effect size test was statistically significant (*Z* = 5.38 *p* < 0.001). The total SMD = −0.55, and the 95% CI = −0.76 to −0.35, as shown in Figure 2.

### 3.5. Publication bias

**FIGURE 3:**
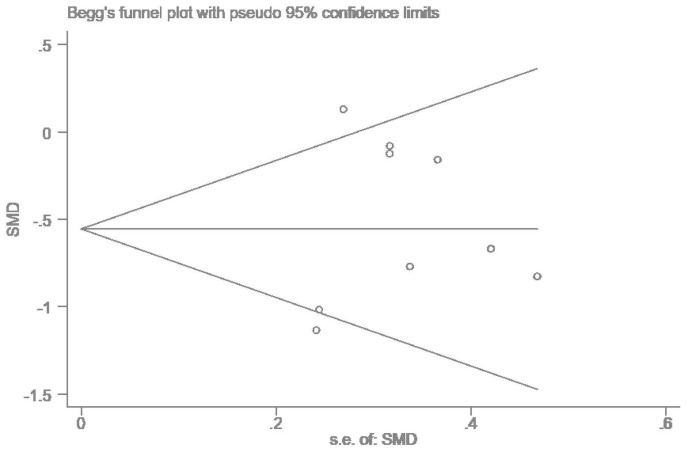
Publication bias graph.

Egger regression analysis was used to test for the publication bias of the nine included articles. The results of Egger’s test–*t* = 0.67, 95% CI = −4.69 to 8.34, *P* = 0.52–demonstrated that there was no publication bias in the included literature (Figure 3).

### 3.6. Subgroup analysis

Subgroup analysis was conducted according to exercise type, exercise frequency, exercise duration, and exercise intensity. In terms of the exercise type, studies were divided into four different groups for analysis: aerobic exercise (included four articles), resistance exercise (2), yoga (1) and other exercises (2). Aerobic exercise, yoga, and other types of interventions were statistically significant (*P* < 0.05) for improving anxiety symptoms, while resistance exercise was not statistically significant (*P* > 0.05). In terms of exercise frequency, the included studies were divided into three groups: high frequency (≥5 times/week) (included one article), moderate frequency (3–4 times/week) (4) and low frequency (1–2 times/week) (4). Both high frequency (SMD = −1.14, 95% CI = −1.61 to −0.66) and moderate frequency (SMD = −0.57, 95% CI = −0.87 to −0.28) were found to be statistically significant for improving anxiety symptoms. Compared with the control group, the effects of the interventions with high and moderate exercise frequencies were statistically significant (*P* < 0.001), but the low exercise frequency was not (*P* > 0.05). The specific results are shown in Table 3.

**TABLE 3:** Summary of meta-analysis results.

Moderator	Subgroup	SMD	95% CI	Subgroup compared with control group		Subgroup comparison	
				***Z***	***P***	***I* ^2^**	***P***
Type of exercise	Aerobics exercise	-0.39	−0.74 to −0.04	2.22	0.027	2.1%	0.382
	Resistance exercise	-0.83	−1.74 to 0.09	1.77	0.077	−	−
	yoga	-0.76	−1.14 to −0.39	3.98	0.000	84.5%	0.011
	Other	-0.50	−0.85 to −0.14	2.76	0.006	90%	0.002
Exercise frequency	High (≥5 times/week)	-1.14	−1.61 to −0.66	4.70	0.000	−	−
	Medium (3–4 times/week)	-0.57	−0.87 to −0.28	3.84	0.000	71%	0.015
	Low	-0.22	−0.57 to 0.13	1.23	0.217	0.00%	0.573
Exercise duration	Short (≤8 weeks)	-0.29	−0.61 to 0.03	1.75	0.080	0.00%	0.563
	Medium (8–14 weeks)	-0.93	−1.32 to −0.54	4.71	0.000	50.0%	0.553
	Long (>14 weeks)	-0.57	−0.92 to −0.22	3.18	0.001	91.8%	0.000
Exercise intensity	High	-0.41	−0.76 to −0.05	2.26	0.024	14.1%	0.322
	Medium	-0.84	−1.12 to −0.57	5.90	0.000	59.0%	0.063
	Low	0.13	−0.40 to 0.66	0.48	0.629	−	−

In terms of duration, the included studies were divided into three groups: short duration (≤8 weeks; 5 articles included), medium duration (8–14 weeks; three articles included) and long duration (>14 weeks; one article included). Compared with the control group, the medium and long durations were found to be statistically significant for anxiety (*P* < 0.001), while the short duration was not (*P* > 0.05). In terms of exercise intensity, the included studies were divided into three groups: high intensity (included four articles), moderate intensity (4) and low intensity (1). High-intensity exercise (SMD = −0.41, 95% CI = −0.76 to −0.05) and moderate exercise intensity (SMD = −0.84, 95% CI = −1.12 to −0.57) were found to be statistically significant for anxiety, but low exercise intensity was not (SMD = 0.13, 95% CI = −0.40 to 0.66). Compared with the control group, the effects of the interventions with high and moderate exercise intensity were statistically significant (*P* < 0.05), while low exercise intensity was not (*P* > 0.05). The findings revealed that exercise interventions with higher intensity or longer durations could be more effective for relieving anxiety symptoms.

## 4. Discussion

In this section, firstly we study the intervention effects of physical activity on anxiety. Then, the effect of different exercise intensities on anxiety will be discussed briefly.

### 4.1. Intervention effects of physical activity on anxiety

The results of this study showed that exercise interventions play a certain role in reducing the symptoms of psychological anxiety in college students. Exercise type, time, and frequency have differing effects on reducing the symptoms of anxiety. In the literature included within this study, most of the interventions consisted of aerobic exercise (Kramer et al., 2005; Marcos de Souza Moura et al., 2015; Yin et al., 2021; Brinsley et al., 2022). Aerobic exercise can be used as an adjunctive therapy to improve the physical health of people with anxiety disorders and has benefits in reducing anxiety symptoms (Bartley et al., 2013). It also helps to prevent heart disease and obesity (Bauman, 2004). With the continuous development of society and the variety of forms of exercise, intervention methods have also diversified, such as resistance training and yoga. The results demonstrated in Table 3 confirmed that, in the subgroup analysis of exercise type, in addition to resistance exercise, aerobic exercise, yoga and other exercise intervention types were statistically significant. Earlier studies have found that low or moderate resistance training might not have an effect on anxiety (Garwin et al., 1997; Focht and Koltyn, 1999). Conversely, high-intensity resistance training might be associated with an increase in state anxiety (Arent et al., 2005). The decrease in anxiety was not significant under resistance exercise (Hill et al., 2019), and resistance exercise was not statistically significant in relieving anxiety symptoms in this study. Aerobic exercise and other types of exercise have greater effects than yoga, indicating smaller group-to-group differences in the included literature.

Different exercise modes, exercise durations and exercise intensities have different effects on improving physical function and have effects on relieving state anxiety and anxiety (Rýzková et al., 2018; Margulis et al., 2021; Ji et al., 2022). A subgroup analysis of exercise frequency revealed that the frequency of exercise is also necessary for anxiety relief. Therefore, in this study, exercise frequency was divided into three levels for analysis to seek the best exercise effect (Russell, 2002; Broman-Fulks et al., 2018). Both the high-frequency exercise group and the medium-frequency exercise group were statistically significant, but the low-frequency exercise frequency was not statistically significant. A higher frequency of exercise in an anxious state can prompt patients to devote more time and energy to exercise so that some anxiety in life or work can be relieved and released during exercise. For anxious states, the need for more treatment options has been emphasized (Ezekowitz et al., 1995). The choice of non-pharmacological interventions can also promote the reduction of anxiety symptoms in college students, and exercise can effectively relieve anxiety symptoms (Carek et al., 2011). Currently, due to the increased psychological burden of learning pressure and social interaction, college students must alleviate their anxiety through a series of exercise interventions or auxiliary therapies (Beiter et al., 2015). Aerobic exercise, such as jogging, as the daily exercise of college students, is easy to popularize and apply in colleges and universities and can be used as an effective intervention measure to prevent college students’ anxiety, stress, and depression (Ibrahim et al., 2013; Stubbs et al., 2017). Exercise can reduce negative emotions and promote the transformation of these negative emotions into positive aspects.

### 4.2. The effect of different exercise intensities on anxiety

Exercise load is a concrete manifestation of exercise time and exercise intensity, and it is a factor that affects the outcome of an exercise. The results of the subgroup analysis showed that medium- and long-term exercise had certain regulatory effects on the intervention effect, and the 95% CI did not contain 0, confirming the significance. Short-term exercise did not have a good effect on awakening body function, and there was no significant difference between the groups. Short-term aerobic exercise has little effect on improving mood and anxiety. This subgroup analysis primarily reported the effect of moderate- or vigorous-intensity physical activity on anxiety, reflecting that a subjective, transient emotional state was also associated with a larger effect size of exercise. A study comparing the effects of swimming, fencing, body conditioning, and yoga classes found that only the yoga treatment group showed that short-term and long-term yoga exercise significantly reduced state anxiety (Berger and Owen, 1988). We also found large differences in exercise interventions due to the studies involving different exercise patterns, times, frequencies, and intensities. The differences in each subgroup were large in the analysis. From the overall subgroup analysis, the effects of lower intensity exercise, lower exercise frequency, and short exercise intervention cycles on anxiety were not statistically significant, and the 95% CI contained 0. The duration, frequency, and intensity of exercise had effects on the experimental intervention. In the process of relieving anxiety, it is not possible to rely solely on physical activity, and it should be used in conjunction with medication, psychological counseling, and psychotherapy to establish a good psychological state.

### 4.3. Limitations

This study had some limitations. First, the included study subjects were university students. Therefore, applying these findings to others in the same age group might be restricted, such as in young people who are already working or young women of childbearing age. Second, the limited number of included studies might have led to some degree of selection bias. Finally, this study included diverse indicators of outcome evaluation, and the heterogeneity test results were relatively large. Therefore, quality control standards for future clinical trials should be based on evidence-based medical standards.

## 5. Conclusion

In summary, aerobic exercise and yoga, as well as other types of exercise, can relieve anxious states. Moderate to high intensity, longer duration periods, aerobic exercise with a higher exercise frequency, and other types of exercise have significant effects on anxiety relief. The current study suggested incorporating appropriate exercise into the lives of people with anxiety symptoms for the significant benefits of alleviating anxiety and developing physical and mental health.

## Data availability statement

The original contributions presented in this study are included in the article/Supplementary material, further inquiries can be directed to the corresponding authors.

## Author contributions

YL was responsible for writing the manuscript. WG was responsible for data collection and analysis. Both authors contributed to the article and approved the submitted version.
