# Supplementary material for: The effects of physical exercise on anxiety symptoms of college students: A meta-analysis
Source: Front Psychol. 2023 Mar 30;14:1136900. doi: 10.3389/fpsyg.2023.1136900 (PMC10100500; doi:10.3389/fpsyg.2023.1136900)
Supplement: Supplementary file 1 [file Data_Sheet_1.docx]

Supplementary Material

**The Effects of Physical Activity on Anxiety Symptoms of College Students: A Meta-Analysis**

Yanru Lin*,Wei Gao

*** Correspondence:**  **Yanru Lin；**46145428@qq.com

# Supplementary Data

**Data:** ((randomized controlled trial[Publication Type] OR randomized[Title/Abstract] OR placebo[Title/Abstract]) AND (("Anxiety"[Mesh]) OR ((((((((Angst[Title/Abstract]) OR (Social Anxiety[Title/Abstract])) OR (Anxieties, Social[Title/Abstract])) OR (Anxiety, Social[Title/Abstract])) OR (Social Anxieties[Title/Abstract])) OR (Hypervigilance[Title/Abstract])) OR (Nervousness[Title/Abstract])) OR (Anxiousness[Title/Abstract])))) AND (("Exercise"[Mesh]) OR (((((((((((((((((((((((((((((Physical Activity[Title/Abstract]) OR (exercises[Title/Abstract])) OR (Activities, Physical[Title/Abstract]))OR (Activity, Physical[Title/Abstract])) OR (Physical Activities[Title/Abstract])) OR (Exercise,

Physical[Title/Abstract])) OR (Exercises, Physical[Title/Abstract])) OR (Physical

Exercise[Title/Abstract])) OR (Physical Exercises[Title/Abstract])) OR (Acute

Exercise[Title/Abstract])) OR (Acute Exercises[Title/Abstract])) OR (Exercise,

Acute[Title/Abstract])) OR (Exercises, Acute[Title/Abstract])) OR (Exercise,

Isometric[Title/Abstract])) OR (Exercises, Isometric[Title/Abstract])) OR (Isometric

Exercises[Title/Abstract])) OR (Isometric Exercise[Title/Abstract]))

OR (Exercise, Aerobic[Title/Abstract])) OR (Aerobic Exercise[Title/Abstract])) OR (Aerobic Exercises[Title/Abstract])) OR (Exercises, Aerobic[Title/Abstract])) OR (Exercise Training[Title/Abstract])) OR (Exercise Trainings[Title/Abstract])) OR (Training, Exercise[Title/Abstract])) OR (Trainings, Exercise[Title/Abstract])))
